# Long term effects of soluble endoglin and mild hypercholesterolemia in mice hearts

**DOI:** 10.1371/journal.pone.0233725

**Published:** 2020-05-29

**Authors:** Barbora Vitverova, Iveta Najmanova, Matej Vicen, Katarina Tripska, Ivone Cristina Igreja Sa, Radek Hyspler, Miguel Pericacho, Petr Nachtigal

**Affiliations:** 1 Department of Biological and Medical Sciences, Faculty of Pharmacy in Hradec Kralove, Charles University, Hradec Kralove, Czech Republic; 2 Centrum for Research and Development, University Hospital, Hradec Kralove, Czech Republic; 3 Renal and Cardiovascular Research Unit, Department of Physiology and Pharmacology, University of Salamanca, and the Biomedical Research Institute of Salamanca (IBSAL), Salamanca, Spain; University of Pennsylvania, UNITED STATES

## Abstract

Soluble endoglin (sEng) released into the circulation was suggested to be related to cardiovascular based pathologies. It was demonstrated that a combination of high sEng levels and long-term exposure (six months) to high fat diet (HFD) resulted in aggravation of endothelial dysfunction in the aorta. Thus, in this study, we hypothesized that a similar experimental design would affect the heart morphology, TGFβ signaling, inflammation, fibrosis, oxidative stress and eNOS signaling in myocardium in transgenic mice overexpressing human sEng. Three-month-old female transgenic mice overexpressing human sEng in plasma (*Sol-Eng*^*+*^ *high*) and their age-matched littermates with low levels of human sEng (*Sol-Eng*^*+*^ *low*) were fed a high-fat diet containing 1.25% of cholesterol and 40% of fat for six months. A blood analysis was performed, and the heart samples were analyzed by qRT-PCR and Western blot. The results of this study showed no effects of sEng and HFD on myocardial morphology/hypertrophy/fibrosis. However, the expression of pSmad2/3 and p-eNOS was reduced in *Sol-Eng*^*+*^ *high* mice. On the other hand, sEng and HFD did not significantly affect the expression of selected members of TGFβ signaling (membrane endoglin, TGFβRII, ALK-5, ALK-1, Id-1, PAI-1), inflammation (VCAM-1, ICAM-1), oxidative stress (NQO1, HO-1) and heart remodeling (PDGFβ, COL1A1, β-MHC). In conclusion, the results of this study confirmed that sEng, even combined with a high-fat diet inducing hypercholesterolemia administered for six months, does not affect the structure of the heart with respect to hypertrophy, fibrosis, inflammation and oxidative stress. Interestingly, pSmad2/3/p-eNOS signaling was reduced in both the heart in this study and the aorta in the previous study, suggesting a possible alteration of NO metabolism caused by six months exposure to high sEng levels and HFD. Thus, we might conclude that sEng combined with a high-fat diet might be related to the alteration of NO production due to altered pSmad2/3/p-eNOS signaling in the heart and aorta.

## 1. Introduction

Endoglin (Eng, CD105, TGFβ receptor III), is a homodimeric transmembrane glycoprotein, that is predominantly expressed in endothelial cells [1]. Eng can be proteolytically cleaved at a juxtamembrane region with subsequent release of its ectodomain, called soluble endoglin (sEng), into the circulation [2, 3].

Increased levels of sEng in plasma are related to cardiovascular based pathologies such as hypercholesterolemia [4], type II diabetes mellitus, hypertension [5], myocardial infarction [6], acute heart failure [7, 8], and preeclampsia [3]. Moreover, the generation of sEng is linked to endothelial injury and high blood pressure [9, 10].

Furthermore, several studies demonstrated the capability of sEng to antagonize membrane endoglin effects via transforming growth factor beta 1 (TGFβ1) cytokine binding [11, 12]. sEng competes with TGFβ1 cytokine for binding to the TGFβ receptor and subsequently influences TGFβ signaling members, including endothelial nitric oxide synthase (eNOS) [3]. eNOS is a key enzyme responsible for nitric oxide (NO) production by endothelium and prevention against endothelial dysfunction [13, 14]. In this context, increased levels of sEng resulted in the development of arterial hypertension in mice [10], which is a pathological basis for the potential development of myocardial hypertrophy, remodeling, fibrosis [15, 16] and impaired myocardial relaxation [17].

Transgenic female mice with high levels of human sEng (*Sol-Eng*^*+*^) were considered an appropriate model to reveal sEng’s effect on the cardiovascular system [10]. It was demonstrated that sEng alone does not affect either functional or morphological parameters in the aorta and heart [18, 19]. In combination with short-term (three months) administration of a high-fat diet, sEng mice induced proinflammatory and oxidative changes in the aorta [20], but surprisingly did not alter morphological parameters in the heart [19]. Previously, it was demonstrated that the combination of high sEng levels and long-term exposure (six months) to a high-fat diet (HFD) resulted in the most pronounced changes in the aorta, which included alteration of endoglin/pSmad2/3/p-eNOS signaling, reduced NO production, and aggravation of endothelial dysfunction [9]. Thus, in this study, we hypothesized that a similar experimental design (the same mice as previously used) would affect the heart morphology and TGFβ signaling with respect to the inflammation, fibrosis, oxidative stress and endothelial dysfunction in myocardium of these mice.

## 2. Materials and methods

### 2.1 Animals and experimental design

Transgenic mice overexpressing human sEng (*Sol-Eng*^*+*^) on the CBAxC57BL/6J background were generated at the Genetically Modified Organisms Generation Unit (University of Salamanca, Spain), as previously described [10]. Three-month-old female mice with high levels of human sEng in plasma (*Sol-Eng*^*+*^ *high*) and their age-matched female transgenic littermates with low levels of human sEng in plasma (*Sol-Eng*^*+*^ *low*) fed a high-fat rodent diet containing 1.25% of cholesterol and 40% of fat (Research Diets, Inc., USA) for the following six months. Female mice were used in all our related experiments in the recent years, in order to make the results at least partially comparable among these studies. Mice with human sEng levels higher than 1000 ng/mL were assigned to *Sol-Eng*^*+*^ *high* group. The animals were kept in controlled ambient conditions in a temperature-controlled room with a 12-h light/dark cycle with constant humidity and had access to tap water and a high-fat diet *ad libitum*. At the age of nine months, the mice were euthanized under general anesthesia induced by a combination of xylazine (10 mg/kg, i.p.) and ketamine (100 mg/kg, i.p.), and blood, tibia and heart samples were harvested for further analysis.

All experiments were carried out in accordance with the standards established in the directive of the European Union (2010/63/EU), and all procedures were approved by the Ethical Committee for the Protection of Animals Against Cruelty at Faculty of Pharmacy, Charles University (Permit Number: 21558/2013-2), and the Bioethics Committee of the University of Salamanca (Permit Number: 006–201400038812). All efforts were made to minimize the suffering of the animals.

### 2.2 Biochemical analysis

Plasma lipoprotein fractions were prepared by using sodium chloride density gradient ultracentrifugation (TL 100, Beckham, Palo Alto, CA, USA). The lipoprotein fractions were separated in the following density ranges: VLDL cholesterol (VLDL-C) < 1.006 g/ml, LDL cholesterol (LDL-C) < 1.063 g/ml and HDL cholesterol (HDL-C) > 1.063 g/ml. The lipoprotein fraction concentrations of cholesterol were measured enzymatically by conventional enzymatic diagnostic kits (Lachema, Brno, Czech Republic) and spectrophotometric analysis (cholesterol at 510 nm, ULTROSPECT III, Pharmacia LKB Biotechnology, Uppsala, Sweden).

### 2.3 Quantitative real-time PCR

Total RNA from heart tissue (myocardium of heart ventricles) was isolated with TRI reagent (Sigma-Aldrich, St. Louis, USA) and was directly reversed transcribed into cDNA using High-Capacity cDNA reverse transcription kit (Life Technologies, Foster City, USA). TaqMan® Gene Expression Master Mix and pre-designed TaqMan® Gene Expression Assay kits for the following genes: membrane Eng (Mm00468256_m1), TGFβ1 (Mm01178820_m1), PDGFβ (Mm00440677_m1), COL1A1 (Mm00801666_g1), NQO1 (Mm01253561_m1), VCAM-1 (Mm01320970_m1), ICAM-1 (Mm00516023_ m1), ALK1/Acvrl1 (Mm00437432_m1), ALK5/TGFβRI (Mm00436964_m1), TGFβRII (Mm03024091_ m1), PAI-1/Serpine1 (Mm00435858_m1), Id1 (Mm00775963_g1), MYH7 (Mm00600555_m1) and GAPDH mouse endogenous control/housekeeping gene (Mm99999915_g1) were provided by Life Technologies (Foster City, USA). Analysis was performed using QuantStudioTM 6. Target gene expression was calculated using delta-delta Ct method as described previously [21]. Expression was normalized to glyceraldehyde 3-phosphate dehydrogenase (GAPDH) expression levels in heart, which were stable across the experimental groups. All data represent fold change over expression in *Sol-Eng*^*+*^ *low* mice.

### 2.4 Western blot analysis

The procedure was performed as previously reported by Rathouska et al. [19]. Protein specific signals in each lane were normalized to GAPDH signal. Specific antibodies are listed in Table 1.

**Table 1:** Primary and secondary antibodies used for Western blot analysis.

Protein	Source	Specification	Dilution (Primary Antibody)	Dilution (Secondary antibody)
COX-2	Abcam	ab1519 Rabbit polyclonal	1:250	1:1,000
GAPDH	Sigma	G8795 Mouse monoclonal	1:10,000	1:20,000
HO-1	Abcam	ab13243 Rabbit polyclonal	1:2,000	1:1,000
p-eNOS Ser1177	Santa Cruz	sc-21871-R Rabbit polyclonal	1:500	1:2,000
pNF-κB	Abcam	ab16502 Rabbit polyclonal	1:500	1:2,000
P-selectin	Abcam	ab59738 Rabbit polyclonal	1:200	1:2,000
pSmad1/5	Cell Signaling	9516S Rabbit monoclonal	1:1,000	1:2,000
pSmad2/3	Cell Signaling	8828S Rabbit monoclonal	1:1,000	1:2,000

### 2.5 Morphometric and histological assessment of heart

Standard morphometric measures were obtained including body and heart weights as well as tibia length. To determine heart hypertrophic phenotype, normalization of body weight and heart weight to tibia length was put into proportion. For the histological evaluation, samples of heart tissue (myocardium of heart ventricles) were immediately fixed in 4% formaldehyde for 24 hours, and then embedded in paraffin. Tissue sections (thickness 7 μm) were prepared using microtome and placed on glass slides. Hematoxylin-eosin staining was performed according to standard techniques. For the collagen detection, Goldner′s green trichrome staining was performed to visualize collagen fibers and connective tissue to reveal possible microscopic changes in the heart.

### 2.6 Statistical analysis

Data are expressed as the mean ± SEM. All analyses were performed using GraphPad Prism 8.0 software (La Jolla, CA, USA). Direct group-group comparisons were carried out using Mann-Whitney test. A value of P ≤ 0.05 was the minimum requirement for a statistically significant difference.

## 3. Results

### 3.1 Plasma lipoprotein profile in *Sol-Eng*^*+*^ mice

**Fig 1:**
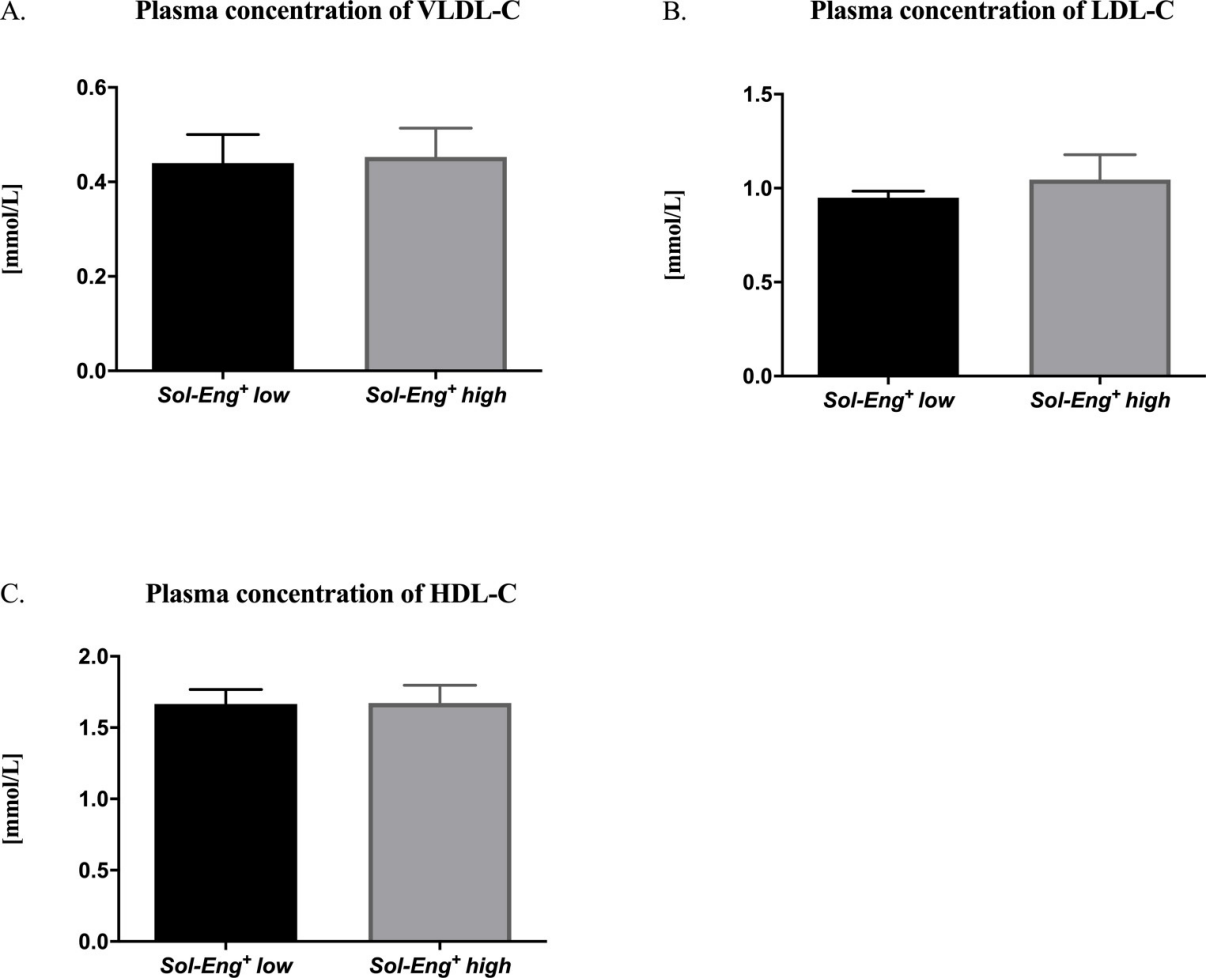
Lipid profile of *Sol-Eng*^*+*^ *mice*. Plasma concentration of VLDL-C (A), LDL-C (B), HDL-C (C) in *Sol-Eng*^*+*^ *high* mice and *Sol-Eng*^*+*^ *low* mice fed high fat diet for six months. Data are shown as mean ± S.E.M., Mann-Whitney test. n = 8 mice per group.

After a six-month high-fat feeding period, plasma samples were used for biochemical analysis of lipid profile. Biochemical analysis showed no differences in VLDL-C concentration (0.45 ± 0.06 vs. 0.44 ± 0.06 mmol/L), LDL-C concentration (1.05 ± 0.13 vs. 0.95 ± 0.04 mmol/L) and HDL-C concentration (1.67 ± 0.12 vs. 1.67 ± 0.10 mmol/L) between *Sol-Eng*^*+*^ *high* mice and *Sol-Eng*^*+*^ *low* mice (Fig 1A, 1B and 1C). However, these mice display mild hypercholesterolemia reflected by significantly increased concentration of total cholesterol in comparison with reference control group of transgenic mice fed a chow rodent diet, as reported previously [9].

### 3.2 Cardiac hypertrophy evaluation in *Sol-Eng*^*+*^ *mice*

**Fig 2:**
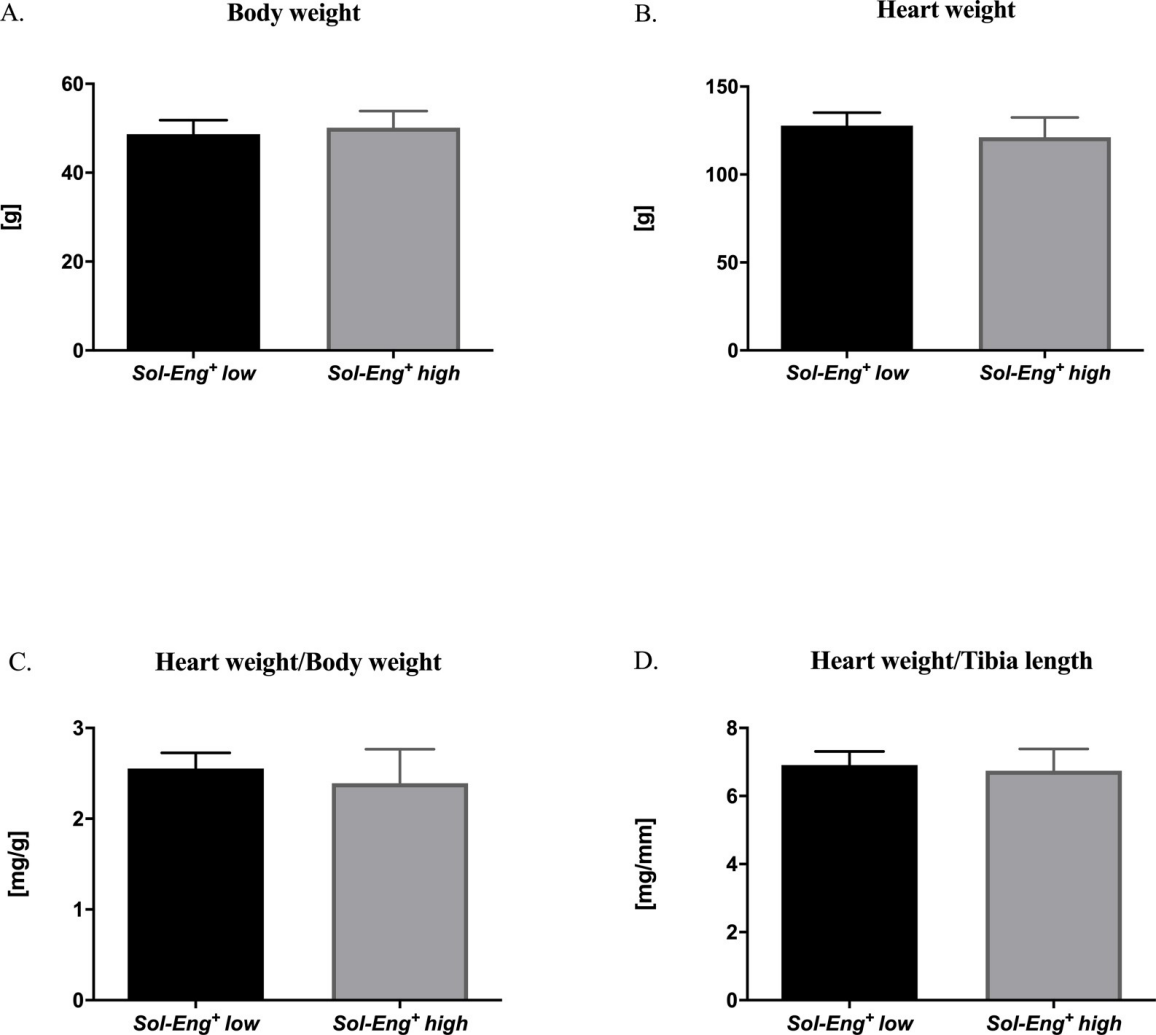
Body weight, heart weight and tibia length of *Sol-Eng*^*+*^ mice. Body weight (A), heart weight (B), heart weight/body weight ratio (C) and heart weight/tibia length ratio (D). Data are shown as mean ± S.E.M., Mann-Whitney test. n = 8 mice per group.

To investigate whether high sEng levels and mild hypercholesterolemia contribute to the development of cardiac hypertrophy, we performed standard morphometric measurement of body weight, heart weight and tibia length. We found no significant difference in body weight (50.14 ± 3.73 vs. 48.70 ± 3.15 g), heart weight (121.20 ± 11.31 vs. 127.90 ± 7.30 mg), heart weight/body weight ratio (2.39 ± 0.37 vs. 2.55 vs. 0.17 mg/g), or heart weight/tibia length ratio (6.75 ± 0.64 vs. 6.91 ± 0.41 mg/mm) in *Sol-Eng*^*+*^ *high* mice compared to *Sol-Eng*^*+*^ *low* mice (Fig 2A, 2B, 2C and 2D).

### 3.3 High sEng levels do not affect TGFβ signaling pathway

Since sEng was shown to interfere with TGFβ1 cytokine, we aimed to evaluate effects of sEng on TGFβ signaling members, including TGFβ downstream activin-like kinase (ALK) receptors and TGFβ receptor II (TGFβRII), in the hearts of these mice.

**Fig 3:**
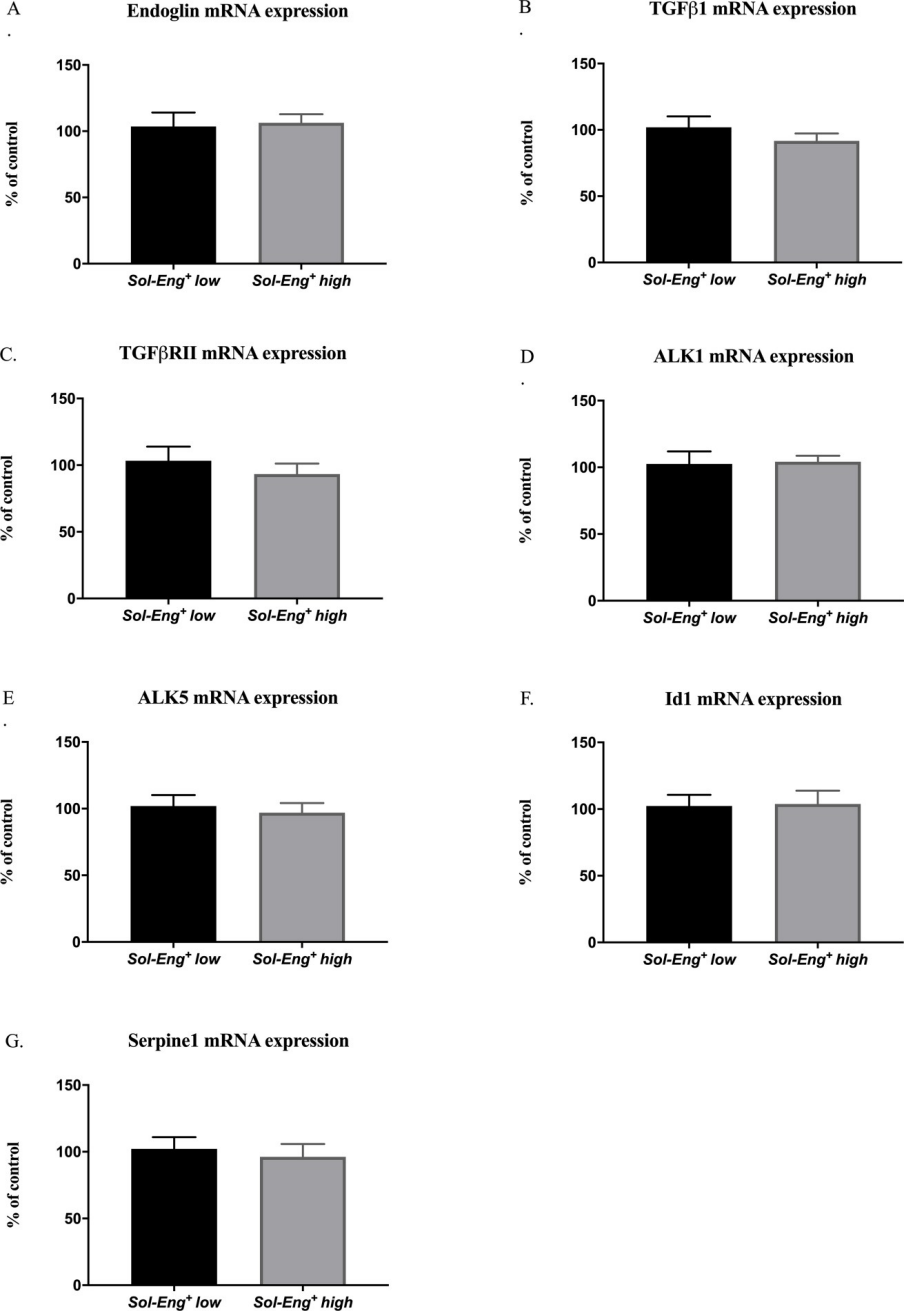
TGFβ signaling pathway in the hearts of *Sol-Eng*^*+*^ mice. mRNA expression of membrane Eng (A), TGFβ1 (B), TGFβRII (C), ALK1 (D), ALK5 (E), Id1 (F) and Serpine1 (G). Data are shown as mean ± S.E.M., Mann-Whitney test. n = 7 mice per group.

Quantitative RT-PCR analysis was performed and no significant differences between *Sol-Eng*^*+*^ *high* and *Sol-Eng*^*+*^ *low* mice in the mRNA expression of membrane Eng (Fig 3A), TGFβ1 cytokine (Fig 3B), TGFβRII (Fig 3C), ALK1 (Fig 3D), ALK5 (Fig 3E), Id1 (Fig 3F) and PAI-1 (gene is encoded and marked as Serpine1, Fig 3G) were observed.

### 3.4 Exposure to high levels of sEng has no significant effect on oxidative stress and remodeling process in the heart

**Fig 4:**
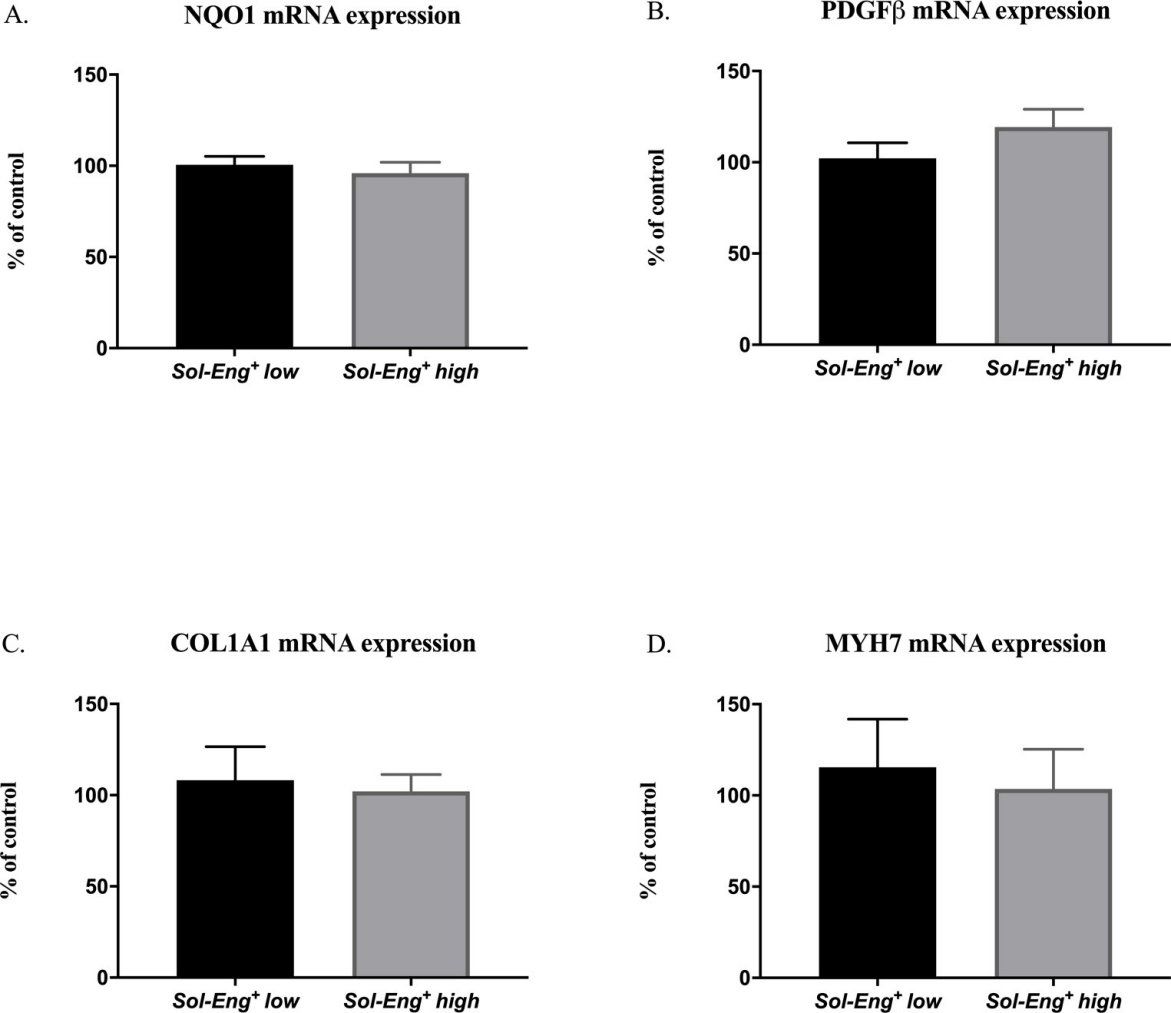
*Sol-Eng^+^ high* mice do not exhibit signs of activated oxidative stress or heart remodeling status. mRNA expression of NQO1 (A), PDGFβ (B), COL1A1 (C) and MYH7 (D). Data are shown as mean ± S.E.M., Mann-Whitney test. n = 7 mice per group.

An excess of ROS results in oxidative stress, which has been shown to participate in cardiac remodeling [22]. Thus, for the assessment of high sEng levels effect on oxidative stress phenotype, we determined gene expression of NADPH quinone acceptor oxidoreductase 1 (NQO1) (Fig 4A) and protein expression of heme oxygenase-1 (HO-1) (Fig 4B).

Development of fibrosis was evaluated by measuring the gene expression of markers potentially involved in cardiac fibrosis. Thus, we performed quantitative RT-PCR analysis of platelet-derived growth factor beta (PDGFβ) gene expression (Fig 4C), type I collagen (encoded by the COL1A1 gene, Fig 4D) and myosin heavy chain beta isoform (β-MHC, encoded by the MYH7 gene, Fig 4E) gene expression.

Quantitative RT-PCR analysis and Western blot analysis revealed no significant differences in the range of the above-mentioned markers reflecting cardiac function between *Sol-Eng*^*+*^ *high* and *Sol-Eng*^*+*^ *low* mice.

### 3.5 High sEng levels do not induce inflammatory phenotype in hearts of *Sol-Eng*^*+*^ *mice*

**Fig 5:**
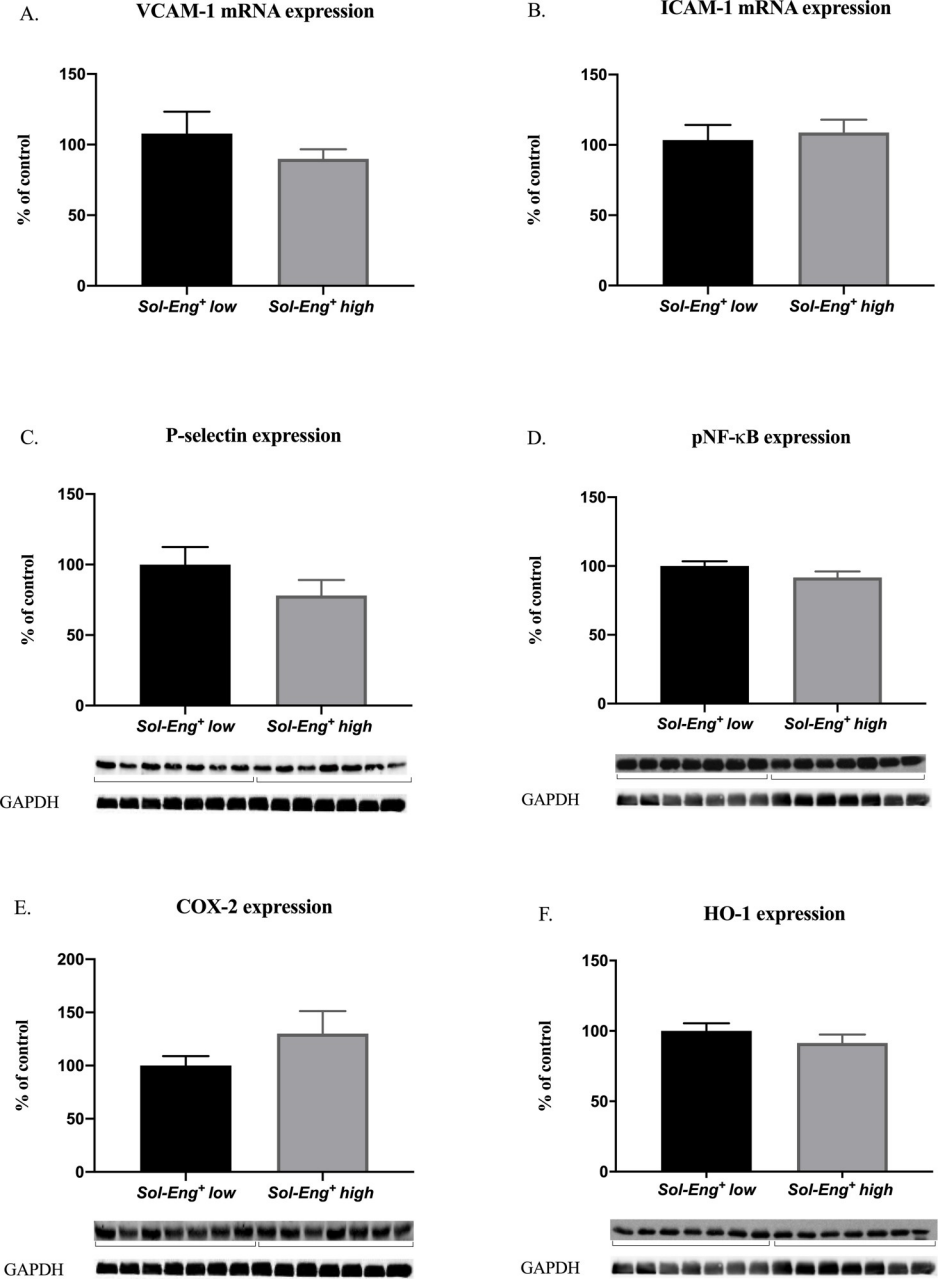
The expression of inflammatory markers in hearts of *Sol-Eng^+^ high* and *Sol-Eng^+^* low mice. mRNA expression of VCAM-1 (A), ICAM-1 (B). The expression of P-selectin (C), pNF-κB (D), COX-2 (E) and HO-1 (F) in total protein extracts from mice hearts. Top: densitometric analysis (control = 100%). Densitometric quantification of immunoreactive bands was recalculated to GAPDH signal. Bottom: representative immunoblots. Data are shown as mean ± S.E.M., Mann-Whitney test. n = 7 mice per group.

For the evaluation of an inflammatory status, we examined gene expression of VCAM-1 (Fig 5A) and ICAM-1 (Fig 5B), and then protein expression of P-selectin (Fig 5C) and phosphorylated (active) nuclear factor kappa B (NF-κB) (Fig 5D). However, no significant changes were observed in the expressions of either VCAM-1, ICAM-1, P-selectin or pNF-κB.

Expression of an inflammation-associated enzyme cyclooxygenase type 2 (COX-2) was measured to reveal a potential mechanism leading to an activation of inflammatory process in the heart tissue [23]. We observed no significant differences in the protein expression of COX-2 (Fig 5E) between the groups as well.

### 3.6 High sEng levels affect pSmad2/3/p-eNOS signaling

**Fig 6:**
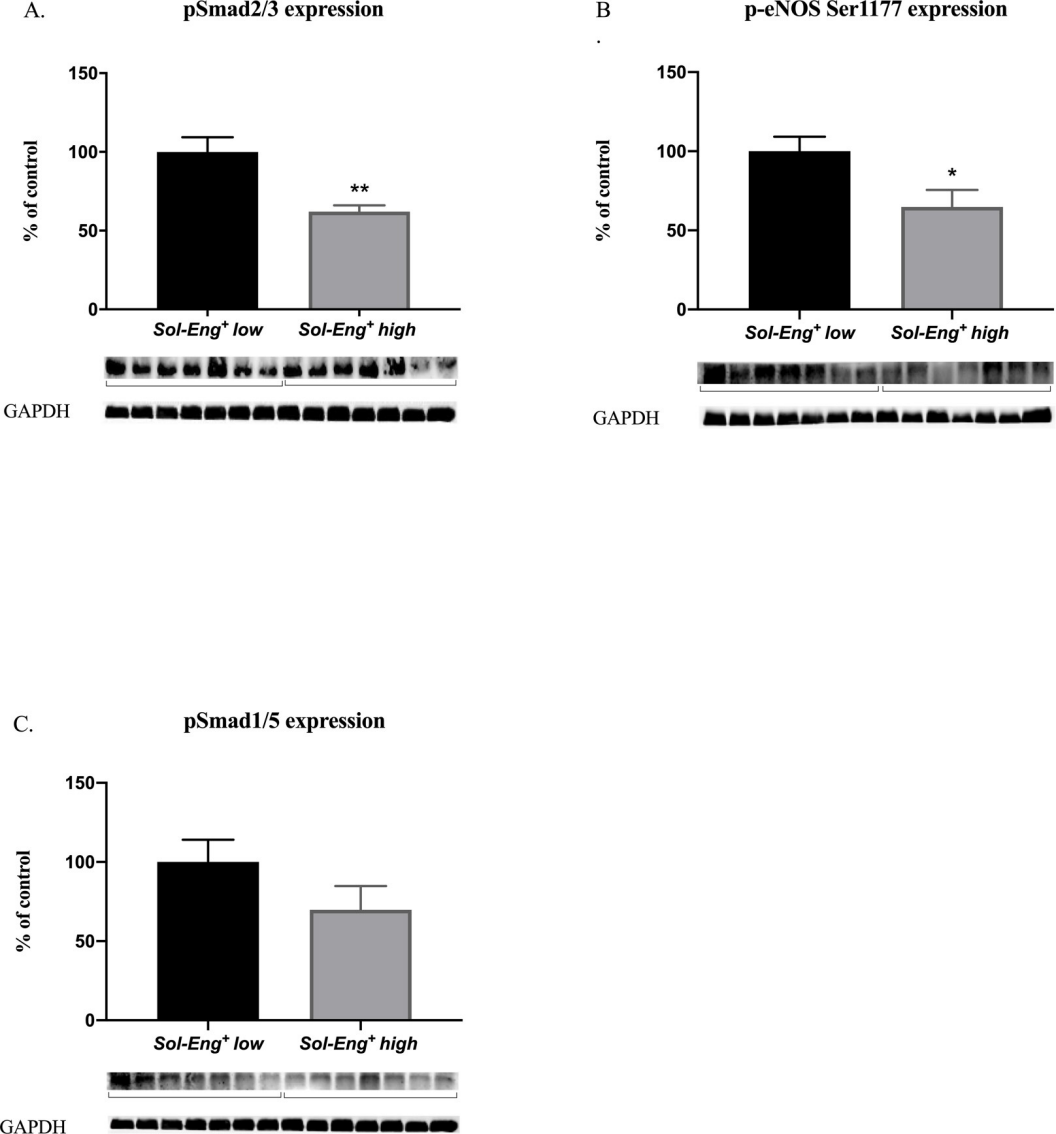
Western blot analysis of Smad expression and signaling in the heart. Expression of pSmad2/3 (A), p-eNOS Ser1177 (B) and pSmad1/5 (C) in total protein extracts from mice hearts. Top: densitometric analysis (control = 100%). Densitometric quantification of immunoreactive bands was recalculated to GAPDH signal. Bottom: representative immunoblots. Data are shown as mean ± S.E.M., Mann-Whitney test, *p≤0.05, **p≤0.01. n = 7 mice per group.

In order to determine whether sEng affects markers of endothelial dysfunction in the heart vessels, we focused on the possible changes of pSmad2/3/p-eNOS signaling. We demonstrated significantly lower expression of phosphorylated (active) form of transcription factor Smad2/3 (pSmad2/3) (to 62%) and phosphorylated eNOS at the serine 1177 position (p-eNOS Ser1177) (to 65%) in *Sol-Eng*^*+*^ *high* mice compared to *Sol-Eng*^*+*^ *low* mice. Also, it is well-established that another signaling pathway might be affected by sEng, thus Smad1/5 expression was assessed (Fig 6C). However, expression of activated transcription factor Smad1/5 (pSmad1/5) did not significantly differ between the groups.

## 4. Discussion

Soluble endoglin (sEng) represents a circulating form of membrane endoglin (Eng), which is crucial for proper function of vascular endothelium. sEng levels are increased in various cardiometabolic disorders that includes atherosclerosis [4, 24], hypercholesterolemia [25], hypertension, type II diabetes mellitus [5] and preeclampsia. Indeed, sEng can be considered as a biomarker in these pathological conditions [11].

However, it has been demonstrated that sEng might interfere/inhibit TGFβ signaling, membrane Eng expression/function, thus affecting the signaling pathway and activities of its signaling members. For instance, sEng was demonstrated to induce eNOS dependent endothelial dysfunction in rats [3]. Moreover, sEng has a crucial role in the development of preeclampsia symptoms [26]. In order to study the impact of high sEng levels *in vivo*, mice expressing human sEng (*Sol-Eng*^*+*^) were generated. *Sol-Eng*^*+*^ mice used in this study show many symptoms of preeclampsia, such as hypertension, small pup size, proteinuria and renal damage [10]. This is currently the only animal model to study the potential effects of sEng *in vivo*. Based on the fact, that these mice develop preeclampsia symptoms, it is highly suggested that human sEng expressed by *Sol-Eng*^*+*^ mice affect mouse TGFβ signaling and/or other mouse signaling systems. Indeed, human and murine endoglin sequences display 71% amino acids sequence identity with almost identical transmembrane and cytoplasmic domains [27].

Recently, we performed several studies focusing on the potential role of sEng in the development of endothelial dysfunction in the aorta and its effect on the heart morphology as well. High levels of sEng alone did not affect the functional and morphological properties of the aorta [18]. Moreover, sEng did not affect the heart with respect to hypertrophy, fibrosis, inflammation and oxidative stress [19]. This situation was changed when high sEng levels were combined with hypercholesterolemia. When *Sol-Eng*^*+*^ mice were exposed to this combination for three months, we demonstrated proinflammatory and oxidative changes in the aorta, however with preserved endothelial function [20]. Surprisingly, the same mice showed no inflammation, fibrosis and/or oxidative stress in the heart [19]. Moreover, it was demonstrated that long-term exposure (six months) to high sEng levels and mild hypercholesterolemia aggravates endothelial dysfunction in mice with alteration of membrane Eng/pSmad2/3/p-eNOS signaling pathway and NO production, suggesting that longer exposure is more “toxic” for blood vessels [9]. Thus, we hypothesized that a similar harmful effect will be detected in the heart with respect to morphology, fibrosis, inflammation, oxidative stress and TGFβ signaling in the same mice.

Firstly, we focused on whether high sEng levels and mild hypercholesterolemia induce the development of cardiac hypertrophy. Thus, we performed standard morphometric measurement of body weight, heart weight and tibia length as well. Tibia length remains immutable throughout life and for this reason is more appropriate for evaluation of cardiac hypertrophy [28, 29]. No significant differences between *Sol-Eng*^*+*^ *high* and *Sol-Eng*^*+*^ *low* mice were detected suggesting no development of hypertrophy due to high levels of sEng and hypercholesterolemia in these mice.

Furthermore, we evaluated potential profibrotic activities in *Sol-Eng*^*+*^ high mice fed by HFD. PDGFβ stimulates cardiac fibroblasts differentiation process into myofibroblasts which leads to highly proliferative and invasive phenotype characterized by remodeling, accumulation of collagen, cardiac fibrosis and hypertrophy [30, 31]. However, no signs of fibrosis either on molecular level (PDGFβ, type I collagen (encoded by the COL1A1 gene) or histological level were detected in both *Sol-Eng*^*+*^ *high* and *Sol-Eng*^*+*^ *low* mice.

Since it was shown that sEng might interfere with TGFβ signaling, we performed an analysis of this signaling in both groups. TGFβ1 cytokine is released by myocardial cells and is generally increased during tissue injury [32–34]. Cellular effects of TGFβ1 cytokine are mediated by its binding to TGFβRI and TGFβRII and subsequent signal transduction through ALK receptors [35]. Membrane endoglin, as an integral part of TGFβ signaling, is involved in modulation of two downstream pathways of TGFβ signaling, specifically ALK1/Id1 and ALK5/PAI-1 [36]. Indeed, we found no significant changes in the expression of involved genes in TGFβ signaling between *Sol-Eng*^*+*^ *high* and *Sol-Eng*^*+*^ *low* mice suggesting that sEng does not interfere with TGFβ signaling in the heart even when hypercholesteremia is present. In fact, these results are in line with recent study showing that sEng more likely binds bone morphogenic protein 9 (BMP-9), forming active molecule, which requires membrane Eng [37] for the proper function. It is of interest to mention that protein expression of membrane Eng was below detection limits in hearts (by Western blot analysis), suggesting that even an effect with respect to BMP-9 in the heart is unlikely.

Since we detected proinflammatory and potential oxidative stress induction in previous studies in aorta, we evaluated several biomarkers of inflammation and oxidative stress in heart in this study as well.

NQO1 plays an important role in cellular protection and protection against oxidative stress by the scavenging of superoxide [38, 39]. HO-1, an inducible enzyme, reflects variety of oxidative challenges and is upregulated during oxidative stress status [40, 41]. In addition, expression of VCAM-1, ICAM-1, P-selectin or pNF-κB are induced during inflammation by several stimuli, and these molecules facilitate transmigration of leukocytes during endothelial dysfunction [42, 43]. However, we did not detect any significant changes of inflammatory and oxidative stress biomarkers between *Sol-Eng*^*+*^ *high* and *Sol-Eng*^*+*^ *low* mice confirming our previous results [19].

On the other hand, the alteration of pSmad2/3/p-eNOS signaling and decreased NO production in aorta was shown recently [9]. In this study, we found the same alteration, showing decreased expression of these molecules in the hearts of *Sol-Eng*^*+*^ *high* mice. This reality might suggest potential development of endothelial dysfunction in the heart circulation. The key question is whether sEng can directly affect pSmad2/3/p-eNOS expression in the hearts of *Sol-Eng*^*+*^ mice. It was demonstrated that sEng can influence vascular development and arteriovenous malformations by modulating angiogenesis, which depends on the expression of membrane Eng in endothelial cells [44]. Moreover, Jerkic et al. showed decreased levels of eNOS in endoglin haploinsufficient mice (Eng^+/-^), which resulted in impaired endothelium-dependent vasodilation in these mice [45]. In addition, Toporsian et al. found that endoglin is an essential component of the eNOS activation complex, stabilizes eNOS protein, and facilitates the association of eNOS [46]. Santibanez et al. clearly showed that endoglin increases Smad2 levels, Smad2 phosphorylation status and its stability, which resulted in increased eNOS expression [47]. Interestingly, undetectable endoglin protein expression was demonstrated in the adult mouse heart when compared to the aorta of CBAxC57BL/6 mouse strain in both studies (this paper and Rathouska et al. [19]). In the light of these information, we might propose that sEng does not significantly interfere with membrane Eng in order to affect pSmad2/3/p-eNOS, at least in the hearts of these mice.

In addition, we cannot evaluate, which blood vessels in the heart show reduced pSmad2/3/p-eNOS expression. On the other hand, pSmad2/3/p-eNOS signaling is altered in both the aorta and the heart suggesting that high levels of sEng combined with hypercholesterolemia interfere with NO production probably via pSmad2/3/p-eNOS signaling cascade in various organs without necessary interference with membrane Eng. However, precise molecular mechanism of sEng effects on pSmad2/3/p-eNOS signaling remains to be elucidated.

There are some, the limitations of the study, which should be mentioned. We did not perform any functional analysis of the heart function. Therefore, we cannot comment impact of altered pSmad2/3/p-eNOS signaling cascade on either systolic or diastolic function or blood circulation in the heart and/or in the coronary circulation. On the other hand, oxidative stress, profibrotic and proinflammatory biomarkers did not differ between *Sol-Eng*^*+*^ *high* and *Sol-Eng*^*+*^ *low* mice. This fact suggests that the hearts were not severely altered by the administration of HFD and high levels of sEng.

In conclusion, the results of this study confirmed that sEng, even combined with a high-fat diet inducing hypercholesterolemia for six months, does not affect the structure of the heart with respect to hypertrophy and cardiac fibrosis. In addition, no effects on TGFβ signaling, inflammation and oxidative stress were detected. Thus, we might propose that the heart is more resistant to the effects of sEng and a high-fat diet (inducing hypercholesterolemia) when compared to aorta. Indeed, pSmad2/3/p-eNOS signaling was reduced in both the heart in this study and the aorta in the previous study, suggesting possible alteration of NO metabolism caused by six months exposure to high sEng levels and high-fat diet. Thus, we might conclude that sEng combined with hypercholesterolemia might be related to the alteration of NO production due to altered pSmad2/3/p-eNOS signaling in the heart and the aorta.

## Supporting information

S1 Raw Images (PDF)
