# Supplementary figures and images for: Long term effects of soluble endoglin and mild hypercholesterolemia in mice hearts
Source: PLoS One. 2020 May 29;15(5):e0233725. doi: 10.1371/journal.pone.0233725 (PMC7259503; doi:10.1371/journal.pone.0233725)

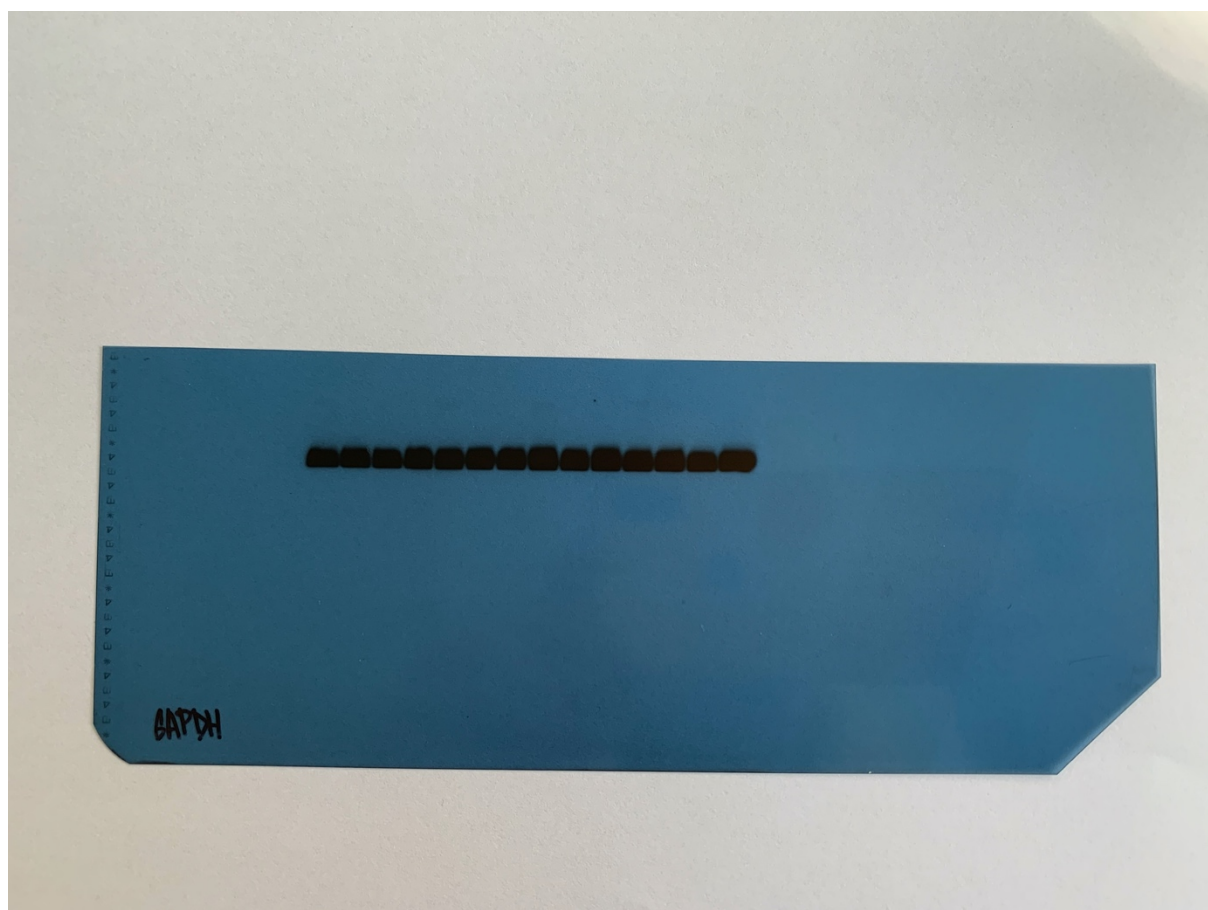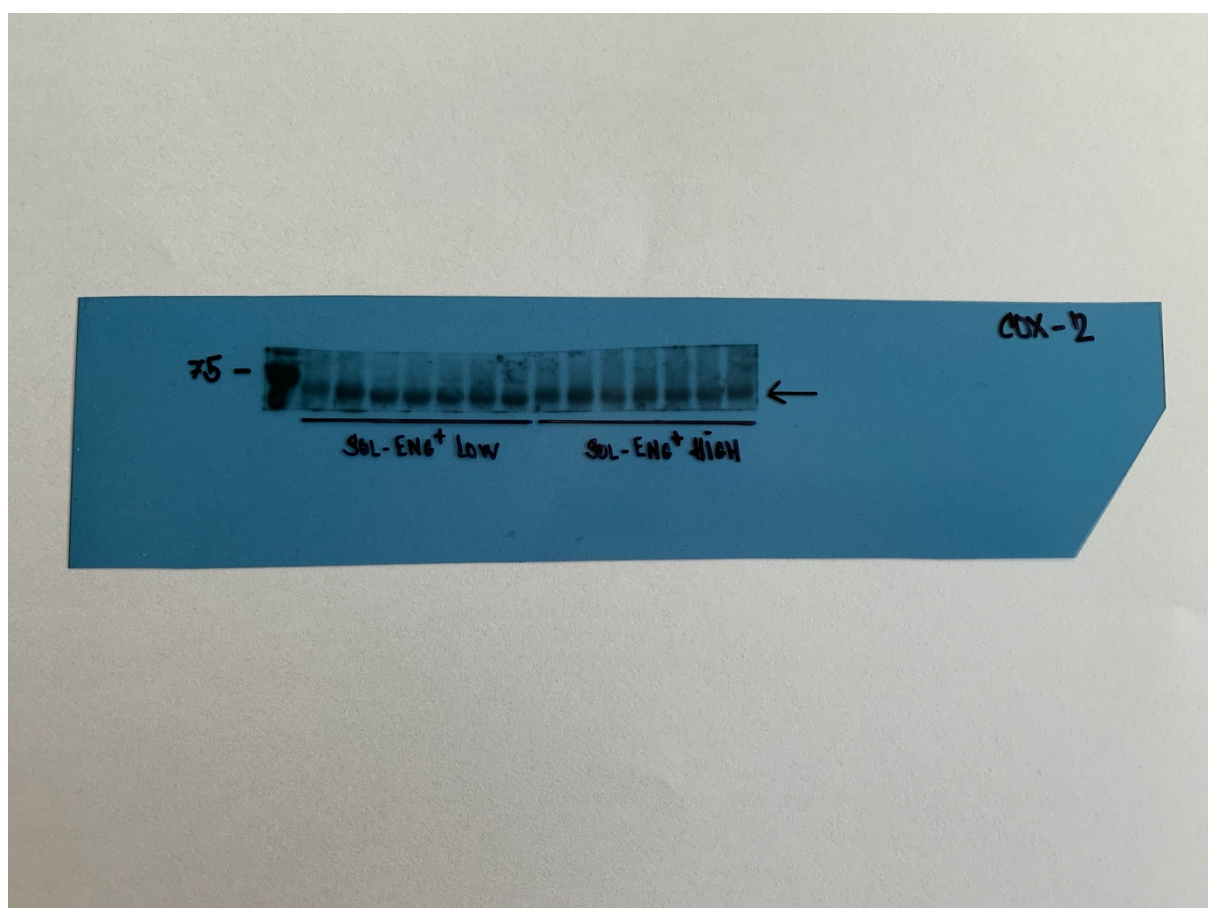

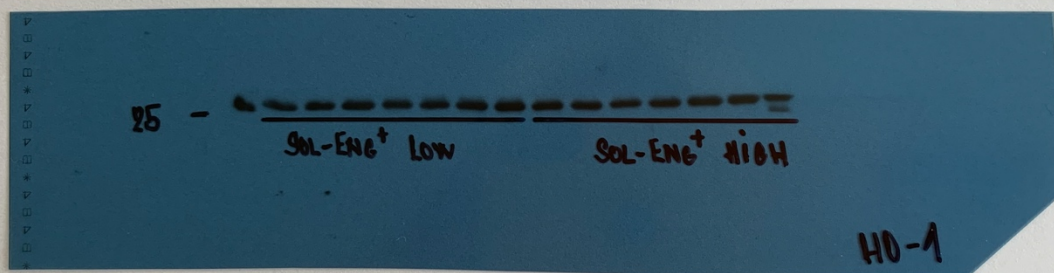

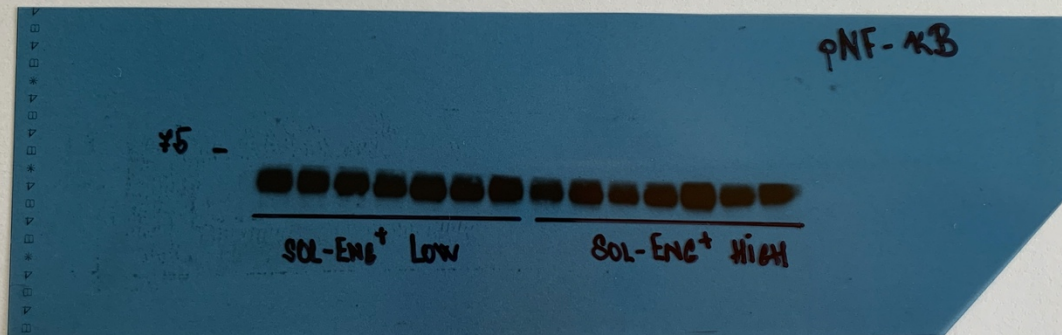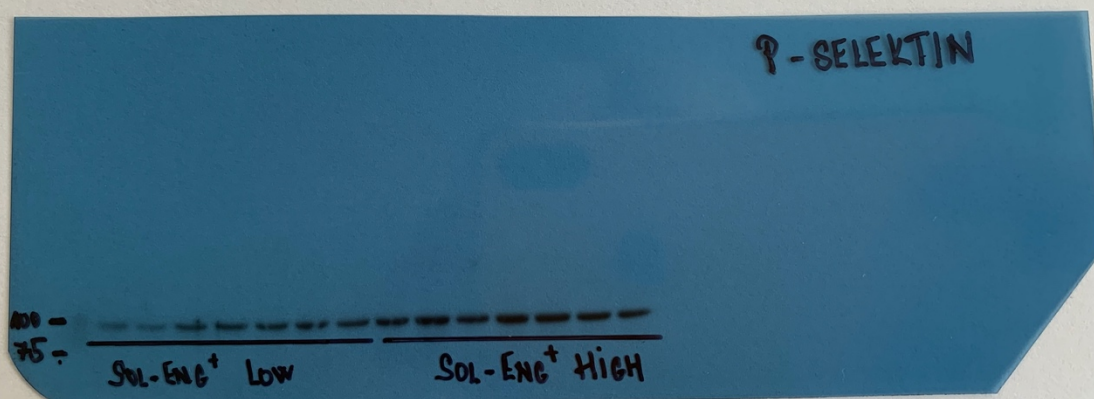

pSHAD 115

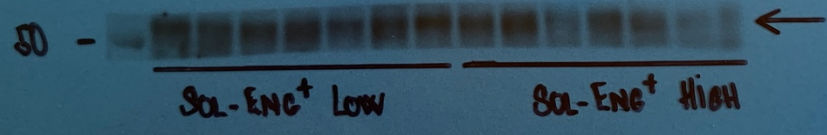

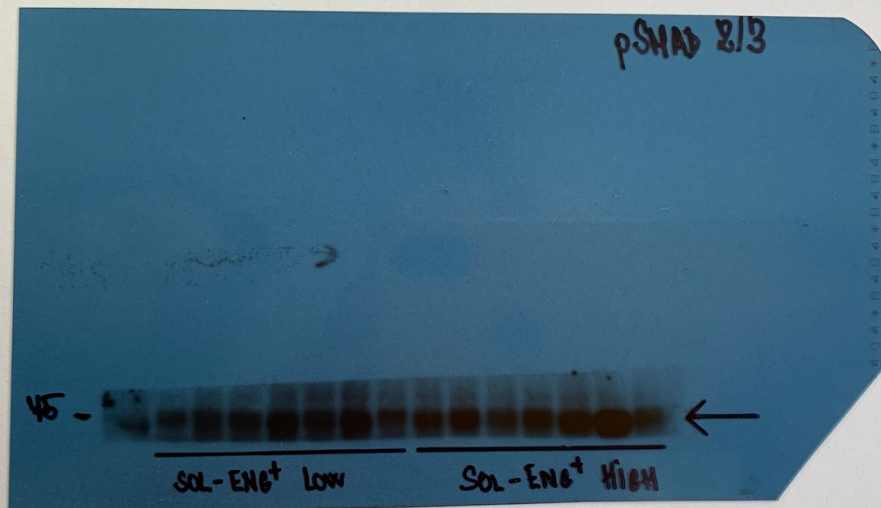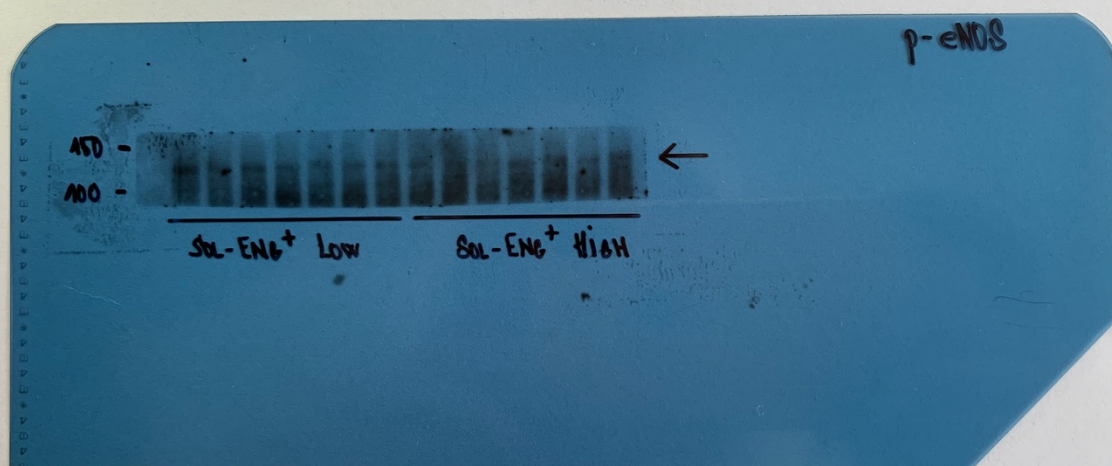

Supplement: S1 Raw Images — (PDF) [file pone.0233725.s001.pdf]
